# Interdisciplinary Perspectives on Dentistry and Sleep Medicine: A Narrative Review of Sleep Apnea and Oral Health

**DOI:** 10.3390/jcm14155603

**Published:** 2025-08-07

**Authors:** Ramona Cioboata, Mara Amalia Balteanu, Denisa Maria Mitroi, Oana Maria Catana, Maria-Loredana Tieranu, Silviu Gabriel Vlasceanu, Eugen Nicolae Tieranu, Viorel Biciusca, Adina Andreea Mirea

**Affiliations:** 1 Department of Pneumology, University of Medicine and Pharmacy, 200349 Craiova, Romania; ramona_cioboata@yahoo.com (R.C.); biciuscaviorel@gmail.com (V.B.); 2 Department of Pneumology, Victor Babes University Hospital, 200515 Craiova, Romania; 3 Department of Pulmonology, Faculty of Medicine, Titu Maiorescu University, 031593 Bucharest, Romania; mara.balteanu@prof.utm.ro; 4 Doctoral School, University of Medicine and Pharmacy, 200349 Craiova, Romania; denisa_maria2@yahoo.com (D.M.M.); oana_cattana@yahoo.com (O.M.C.); 5 Department of Obstetrics and Gynecology, Emergency County Hospital Craiova, 200642 Craiova, Romania; 6 Department of Microbiology, “Carol Davila” University of Medicine and Pharmacy, 050474 Bucharest, Romania; 7 Department of Internal Medicine-Cardiology, University of Medicine and Pharmacy Craiova, 200642 Craiova, Romania; eugen.tieranu@umfcv.ro; 8 Department of Oral-Dental Prevention, University of Medicine and Pharmacy, 200349 Craiova, Romania; adinaturcu14@yahoo.com

**Keywords:** obstructive sleep apnea syndrome, OSAS, dental manifestations, oral appliance therapy, dental management

## Abstract

Obstructive sleep apnea syndrome (OSAS) is a prevalent disorder with significant systemic and oral health consequences. This narrative review synthesizes the current knowledge on the interplay between dental health and sleep apnea, highlighting the expanding role of dentists in the screening, early detection, and management of OSAS. Validated questionnaires, anatomical assessments, and anthropometric measurements have enhanced dentists’ capacity for early screening. However, knowledge and training gaps remain, particularly in low- and middle-income countries. Dentists are uniquely positioned to identify anatomical and oral risk factors, facilitate referrals for diagnosis, and provide therapeutic interventions such as oral appliance therapy. Interdisciplinary collaboration between dental and medical professionals is essential to improve early detection, treatment outcomes, and patient quality of life. Enhancing education, standardizing protocols, and integrating dentists into multidisciplinary care pathways are critical steps for advancing the management of sleep apnea.

## 1. Introduction

Sleep apnea syndrome is a prevalent disorder, affecting an estimated 1 billion adults worldwide, and is marked by repeated episodes of breathing cessation or reduction during sleep, resulting in fragmented sleep and decreased oxygen saturation [1,2,3]. Obstructive sleep apnea syndrome (OSAS), which accounts for approximately 85–90% of cases, is defined by recurrent upper airway collapse during sleep and is manifested by symptoms including loud snoring, witnessed apneas, and excessive daytime sleepiness.

Major risk factors for OSAS include obesity, male sex, older age, and craniofacial abnormalities. Untreated OSAS significantly increases the risk of hypertension, cardiovascular disease, stroke, metabolic syndrome, and neurocognitive impairment, and is associated with a higher incidence of motor vehicle accidents due to daytime sleepiness [1,3]. Polysomnography remains the diagnostic gold standard, and continuous positive airway pressure (CPAP) is the primary treatment; however, adherence can be challenging. Alternative treatments include oral appliances, weight loss, and surgery [2,3].

Emerging evidence highlights a strong link between sleep apnea and poor oral health. Up to 60% of OSAS [4] patients have moderate to severe periodontal disease, likely related to systemic inflammation and frequent mouth breathing, which worsens xerostomia and caries risk [5].

Changes in palatal structure, malocclusion, and occlusal alterations related to oral appliance therapy (OAT) are frequently observed in OSAS patients, highlighting the importance of ongoing dental monitoring [5,6]. Dentists can readily identify key oral risk factors for OSAS during routine examinations, including retrognathia, high-arched palate, and enlarged tongue. Additionally, OSAS patients often have a higher prevalence of dental caries, pain, and chewing discomfort, partly due to mouth breathing and dry mouth [7,8].

Given these associations, dentists play a critical role in screening, early detection, and management of OSAS, as well as monitoring for oral side effects of therapy [8]. Dental sleep medicine is rapidly evolving, emphasizing the need for enhanced collaboration between dental and medical professionals [5,6,9].

In recent years, the integration of dental and medical disciplines has become increasingly vital for comprehensive management of OSAS, driven by advancements in diagnostic tools, digital technology, and innovative therapeutic options. Emerging technologies, including three-dimensional (3D) imaging, digital scanning, and telemedicine, have significantly enhanced the diagnostic accuracy and patient monitoring capabilities of dental practices [10,11]. Novel treatments, including digitally designed and precisely fabricated oral appliances, custom mandibular advancement devices produced using computer-aided design/computer-aided manufacturing (CAD/CAM) technologies, and telehealth-supported patient follow-up, are reshaping clinical practices and improving patient adherence and outcomes [12,13]. Embracing interdisciplinary collaboration through these technological innovations not only streamlines diagnostic processes but also promotes personalized care, highlighting the expanding and indispensable role of dental professionals in multidisciplinary teams dedicated to the effective management of OSAS [11,14].

This narrative review summarizes current knowledge on the relationship between dental health and sleep apnea syndrome, including pathophysiology, oral manifestations, diagnostic strategies, and the expanding role of dentists in interdisciplinary care and research.

## 2. Search Strategy

**Figure 1:**
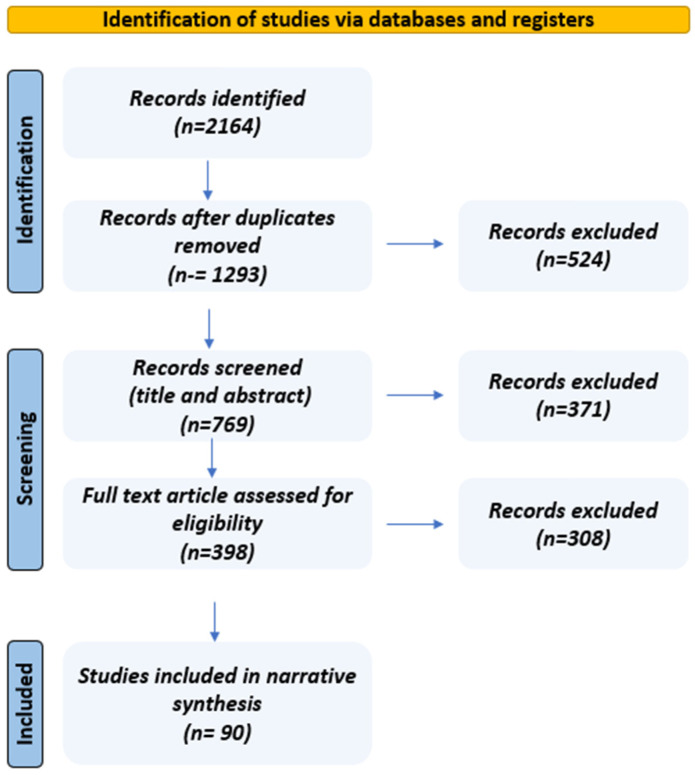
PRISMA flow diagram for literature search.

To enhance transparency and reproducibility of our narrative review, we conducted a targeted literature search of PubMed, Embase, Scopus, and Web of Science databases for relevant articles published from 2005 to 2025. Keywords included combinations of terms including “obstructive sleep apnea,” “OSAS,” “oral health,” “dentistry,” “oral appliance therapy,” “dental sleep medicine,” “craniofacial abnormalities,” and “interdisciplinary care.” Only English-language original research articles, systematic reviews, meta-analyses, and narrative reviews were included in this review. Studies with insufficient details or those published outside the stated publication period were excluded. After removing duplicates, abstracts and titles were screened, followed by full-text reviews to select the relevant literature. To enhance transparency, we included a modified PRISMA flow diagram to illustrate the study selection process (Figure 1). This narrative review synthesizes findings based on relevance and applicability to clinical practice, rather than systematic data extraction. Therefore, no formal quality assessment or quantitative synthesis was performed.

## 3. Pathophysiology of Sleep Apnea

**Figure 2:**
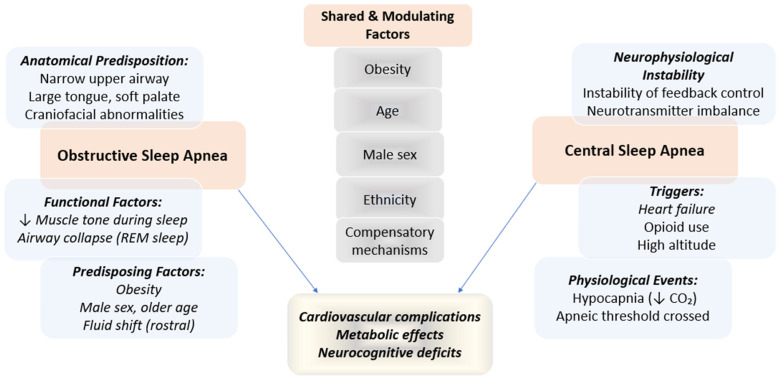
Pathophysiology of obstructive and central sleep apnea.

The pathophysiology of sleep apnea is multifactorial and involves an interplay between anatomical, neuromuscular, and ventilatory control mechanisms. In patients with OSAS, recurrent upper airway collapse during sleep is primarily driven by anatomical airway narrowing and a loss of pharyngeal muscle tone, particularly during REM sleep [15,16]. Structural factors, including craniofacial abnormalities and soft tissue accumulation, combine with neuromuscular deficits to determine airway patency and disease severity [15,17]. Sleep-induced reductions in upper airway dilator muscle activity impair the ability to counteract negative inspiratory pressures, facilitating airway occlusion during inspiration [15,17] (Figure 2).

Central sleep apnea (CSA) arises from transient reductions or absence of central respiratory drive, leading to inadequate ventilation. Instability in ventilatory control, often conceptualized as “loop gain,” is a key factor in CSA pathogenesis. Common triggers and subtypes of CSA include heart failure, chronic opioid use, and high-altitude exposure. In some cases, sleep-induced hypocapnia (low CO_2_) reduces the drive to breathe below the “apneic threshold,” resulting in central apneas. Additionally, neurochemical imbalances, including dysfunction in GABAergic and glutamatergic pathways, may impair activation of brainstem respiratory centers and contribute to CSA development [18,19].

Obesity, age, and sex significantly influence the pathophysiology and severity of sleep apnea. Obesity increases upper airway collapsibility and ventilatory control instability (“loop gain”), while male sex and older age are associated with greater OSAS risk due to similar mechanisms. Ethnic differences in craniofacial structure and other traits may also affect individual susceptibility to sleep apnea. Moreover, the degree to which an individual can compensate for airway obstruction or ventilatory instability varies, influencing disease severity and treatment response [15,17]. Severe sleep apnea, regardless of type, is strongly associated with systemic hypertension, impaired glucose regulation, and increased risk of cardiovascular disease, primarily due to intermittent hypoxemia and heightened sympathetic nervous system activation. Repeated episodes of nocturnal hypoxemia and sleep fragmentation can also cause neurocognitive impairment, leading to daytime sleepiness, memory deficits, and impaired concentration [20].

Regardless of type, the resulting intermittent hypoxemia and sleep fragmentation have widespread systemic effects, including sympathetic activation, metabolic dysregulation, and neurocognitive impairment. Individual susceptibility and clinical expression are shaped by the balance between these anatomical and physiological factors, underscoring the need for personalized approaches to diagnosis and treatment.

## 4. Oral/Dental Manifestations of Sleep Apnea

OSAS has significant oral health implications, many of which can serve as early diagnostic indicators. Common oral manifestations of OSAS include structural abnormalities like high-arched palate, malocclusion, and retrognathia, readily identifiable by dentists during routine examinations. OSAS patients exhibit at least one associated oral manifestation, most notably bruxism, affecting 37.1% of patients, significantly higher than in the general population and frequently resulting in tooth wear and temporomandibular discomfort [21]. Mouth breathing, reported in over half of OSAS patients, contributes to persistent xerostomia (dry mouth), found in 45–60% of adults and approximately 50% of pediatric cases [22], subsequently elevating dental caries risk and oral discomfort [23,24]. Additionally, OSAS is strongly associated with periodontal disease; around 60% of adults with moderate-to-severe OSAS have significant periodontitis, roughly twice the prevalence seen in the general population. Systematic reviews consistently report that individuals with OSAS have a 1.6 to 2.5 times greater likelihood of developing periodontitis compared to unaffected populations, emphasizing the significant inflammatory interplay between these conditions [25,26,27].

The association between the severity of OSAS and periodontal disease is intricate. Some studies suggest a stronger correlation in cases of mild-to-moderate OSAS, while others indicate that more severe forms of OSAS are associated with increased periodontal damage. Both OSAS and periodontal disease exhibit shared inflammatory pathways, particularly characterized by elevated levels of inflammatory markers, including interleukin-6 (IL-6) in saliva [28]. The systemic inflammation characteristic of OSAS, triggered by intermittent hypoxia and oxidative stress, likely exacerbates periodontal tissue damage and bone loss. These interconnected mechanisms highlight the complex interplay between oral and systemic health in OSAS patients [29,30].

Temporomandibular joint disorders (TMD), characterized by jaw pain and joint dysfunction, are notably prevalent in OSAS patients, affecting up to 25% [7,31]. Oral appliance therapy, commonly used for OSAS management, can result in dental occlusal and skeletal changes in about 20–30% of long-term users, underscoring the importance of regular dental follow-up [7,23,24]. Pediatric OSAS patients frequently exhibit abnormal dental arch development, with a high-arched palate and malocclusion occurring in 40–50% of cases, nearly twice the prevalence observed in the general pediatric population. These oral manifestations underline the critical role dental professionals play in early OSAS identification, management, and interdisciplinary collaboration to optimize both oral and systemic health outcomes [32].

## 5. The Dentist’s Role in Screening and Diagnosis

### 5.1. Screening for OSAS

**Figure 3:**
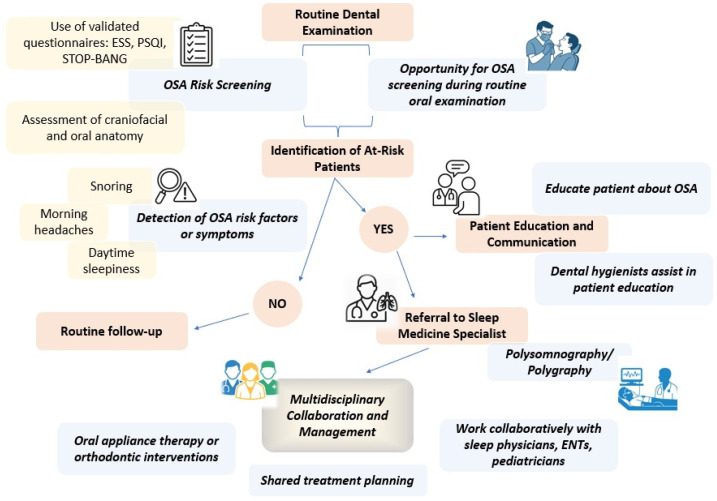
The dentist’s role in screening and diagnosis of OSAS.

Dentists occupy a pivotal role in the early detection and management of OSAS, capitalizing on their frequent patient interactions and the unique opportunity to assess oral and craniofacial structures during routine examinations. Evidence suggests that dentists routinely employ validated screening instruments, including the Epworth Sleepiness Scale (ESS) and the Pittsburgh Sleep Quality Index (PSQI), in conjunction with anatomical assessments, to identify patients exhibiting features suggestive of OSAS, including retrognathia, high-arched palate, enlarged tonsils or tongue, elevated Mallampati score, bruxism, and other signs of airway obstruction [33,34,35,36]. In addition, anthropometric measures like body mass index (BMI) and neck circumference are increasingly recognized as valuable risk indicators, particularly in select populations, notably athletes. The integration of these screening modalities into dental practice not only facilitates the early identification of individuals at risk for OSAS, thereby improving patient outcomes and preventing serious complications, but also underscores the essential role of dentists in multidisciplinary care models (Figure 3).

Dentists are encouraged to refer suspected cases to sleep specialists for definitive diagnosis and collaborate with broader healthcare teams to ensure comprehensive management [5,31]. Notably, integrating systematic dental screenings into routine visits offers substantial benefits for both pediatric and adult populations, enabling early identification of craniofacial anomalies and related OSAS risk factors across diverse age groups. Nonetheless, persistent challenges, particularly inadequate training among dental professionals, underscore the critical need for enhanced educational initiatives and standardized screening protocols within dental practice. Addressing these barriers is essential to optimize early detection, improve referral pathways, and ultimately enhance patient outcomes through interdisciplinary collaboration [37]. Collectively, current evidence positions dental professionals as integral contributors to the early screening and coordinated management of sleep apnea syndrome, with significant implications for both clinical practice and patient outcomes.

**Figure 4:**
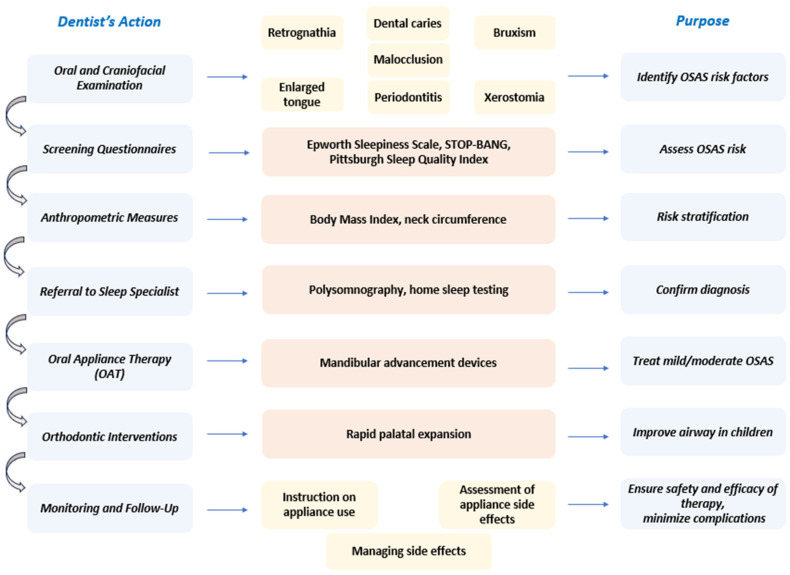
Observations and interventions by dentists in the diagnosis and management of OSAS.

In particular, dentists are uniquely positioned to play a vital role in the early detection and management of OSAS, frequently serving as the first healthcare professionals to recognize signs of the disorder during routine dental examinations [31]. During routine dental visits, dentists are uniquely positioned to perform systematic assessments for anatomical and oral risk factors associated with OSAS. Through detailed oral examinations, they can identify key features, including retrognathia, high-arched palate, enlarged tongue or tonsils, malocclusion, bruxism, periodontal disease, and signs of mouth breathing or xerostomia craniofacial abnormalities that are associated with an increased risk of OSAS. The use of validated screening tools, including the STOP-BANG questionnaire, alongside targeted inquiries about symptoms like snoring, excessive daytime sleepiness, and morning headaches, enables dentists to flag patients who may require further evaluation [38]. Dental hygienists also contribute significantly by educating patients about OSAS risks and facilitating referrals for specialist assessment. When OSAS is suspected, dentists are responsible for referring patients to sleep medicine specialists for definitive diagnosis, typically conducted via polysomnography, and collaborating closely with otolaryngologists and sleep physicians to ensure comprehensive, multidisciplinary care [39,40]. In terms of treatment, dentists are well-equipped to provide OAT, most notably mandibular advancement devices, for patients diagnosed with mild to moderate OSAS or those who are intolerant to CPAP therapy. Early orthodontic intervention, including rapid palatal expansion, may be beneficial for pediatric patients, improving airway structure and reducing the severity of OSAS [40,41]. Ongoing dental and medical follow-up is essential to monitor therapeutic outcomes and make necessary adjustments [42,43] (Figure 4).

Nevertheless, research indicates variability in dentists’ knowledge of OSAS diagnosis and management, emphasizing the need for targeted education and continuous professional development to maximize their impact in this domain [37,44]. Collectively, dentists’ involvement in the identification, referral, and ongoing management of OSAS can substantially improve patient outcomes and overall quality of life.

### 5.2. Referral Pathways to Sleep Medicine Specialists

Efficient referral pathways to sleep medicine specialists are fundamental for the timely diagnosis and management of sleep disorders, including OSAS. However, several significant barriers remain, including limited awareness of sleep disorders among healthcare providers, lack of clarity in referral protocols, and financial or insurance-related constraints. Collectively, these factors may hinder or delay access to specialist care [45]. Facilitators of effective referral include clearly defined roles within the healthcare team, streamlined referral processes, and improved provider knowledge regarding sleep disorders. Innovative models such as electronic consults (eConsults), where primary care providers refer cases electronically for asynchronous specialist review, have demonstrated a marked reduction in specialist response times, from a median of three months to just two days, thereby greatly enhancing access to care [46]. Furthermore, team-based approaches that involve nurses and administrative staff in the triage and coordination of referrals have been shown to increase efficiency, patient access, and overall staff satisfaction, particularly when supported by decision support tools for safe triage. Clinical guidelines underscore the importance of guideline-based referrals, including recommendations to refer patients with OSAS who are intolerant to positive airway pressure (PAP) therapy to sleep or bariatric surgeons, taking into account BMI and individual patient preferences [47]. Additionally, standardized pre-operative referral pathways for surgical patients suspected of OSAS can reduce waiting times and enhance perioperative safety [48,49]. In conclusion, the development of effective, well-defined referral systems supported by provider education, innovative technologies, and adherence to clinical guidelines remains critical to ensuring timely and appropriate care for patients with sleep disorders.

### 5.3. Use of Home Sleep Testing in Dental Practice

Although polysomnography (PSG) remains the gold standard for diagnosing OSAS in adults with suspected moderate to severe disease, recent developments in artificial intelligence (AI) are transforming sleep diagnostics [50]. Advanced remote monitoring and analytical technologies now enable modern wearable and wireless home sleep testing (HST) devices to capture comprehensive physiological data, including EEG, ECG, and SpO_2_, which can be analyzed by AI algorithms through cloud-based platforms. These AI-driven analyses autonomously stage sleep, identify apneas, and flag abnormalities with diagnostic accuracy, matching or exceeding traditional manual scoring. Innovations, including camera-based remote photoplethysmography (PPG) and IoT-enabled systems, further support real-time, contactless monitoring, significantly enhancing accessibility and efficiency of sleep assessments [51,52].

In dental practice, although HST is gaining recognition as a valuable adjunctive tool for screening OSAS, direct implementation by dentists remains limited. Dentists predominantly utilize validated questionnaires (e.g., Epworth Sleepiness Scale, Pittsburgh Sleep Quality Index) alongside anatomical and craniofacial evaluations, occasionally supplemented by overnight pulse oximetry [33,37]. Typically, dental professionals refer patients requiring HST or comprehensive polysomnography to sleep medicine specialists, underscoring their integral role in multidisciplinary care pathways. As AI-driven home sleep monitoring technology evolves, greater integration within dental settings could expand the role of dentists in early OSAS detection and streamline interdisciplinary management approaches [33].

### 5.4. Dental Management and Oral Appliance Therapy

Dental management of OSAS increasingly centers on OAT, particularly for patients who are unable to tolerate CPAP therapy. Dentists play a crucial role in the screening, fitting, and ongoing monitoring of these devices as integral members of multidisciplinary care teams.

Oral appliances, especially mandibular advancement devices (MADs), have demonstrated efficacy in treating mild to moderate OSAS and are considered for select cases of severe OSAS when CPAP is not tolerated [53]. The World Sleep Society endorses the European Respiratory Society’s guideline on non-CPAP therapies for obstructive sleep apnea, recommending bariatric surgery, mandibular advancement devices, myofunctional therapy, and carbonic anhydrase inhibitors. These evidence-based recommendations further underscore the importance of dentists in the multidisciplinary management of OSAS, especially in the selection and monitoring of OAT [54]. OAT has been shown to improve the apnea–hypopnea index and increase airway space in both adult and pediatric populations, and is associated with high levels of patient compliance [55,56]. While OAT is generally less effective than CPAP in reducing OSAS severity, its greater acceptance and adherence among patients may lead to comparable real-world outcomes. Mechanistically, most oral appliances function by advancing the lower jaw, thereby preventing airway collapse during sleep. Both custom-made and non-custom devices are available, with custom appliances providing superior fit and the ability to titrate for optimal results [55,57].

Recent advancements in OAT, including solutions for edentulous patients, have broadened therapeutic options for OSAS. Although generally well-tolerated and associated with improved quality of life [58,59], OAT can lead to side effects including jaw discomfort, tooth movement, and mild occlusal alterations, including decreased overbite, reduced overjet, and subtle shifts in incisor position. While these changes are typically minor and manageable with consistent dental monitoring, they highlight the importance of regular follow-up. A notable challenge remains the significant knowledge gap among dentists and healthcare providers, reflecting limited formal training in OAT. Consequently, achieving optimal patient outcomes depends heavily on enhanced interdisciplinary collaboration, targeted professional education, and the development of standardized clinical protocols to guide appropriate use and monitoring of oral appliance therapy [37,43].

### 5.5. Emerging Technologies and Innovations in Dental Sleep Medicine

Dental sleep medicine is rapidly evolving into a broader, technology-driven, and interdisciplinary specialty. The scope of this field has expanded significantly beyond traditional management of OSAS and snoring, now encompassing conditions including sleep bruxism, orofacial pain, xerostomia, hypersalivation, gastroesophageal reflux disease, and burning mouth syndrome [60,61]. This shift reflects a more comprehensive approach to dental and orofacial conditions linked to sleep, emphasizing the need for multidisciplinary collaboration among dentists, physicians, and other healthcare professionals [62].

Technological advancements have played a pivotal role in reshaping clinical practice and enhancing patient care. Three-dimensional (3D) craniofacial imaging and computer-aided design/computer-aided manufacturing (CAD/CAM) technologies enable dentists to customize oral appliances with unprecedented precision, significantly improving patient comfort, adherence, and clinical outcomes. Concurrently, consumer-friendly wearable sleep-testing devices and smartphone applications are facilitating dentists’ direct involvement in preliminary screening and continuous patient monitoring. Although promising, the widespread implementation of these wearable technologies remains limited due to regulatory, training, and standardization challenges [63,64].

AI represents another transformative innovation rapidly integrated into dental sleep medicine. AI-driven diagnostic tools offer potential for precise and rapid identification of anatomical risk factors, enhancing early detection of OSAS and related conditions. Furthermore, AI-supported telemedicine platforms have expanded remote healthcare delivery capabilities, particularly accelerated by the COVID-19 pandemic [63,65].

Despite these technological breakthroughs, effective clinical integration requires addressing significant barriers, including gaps in dentist training and regulatory constraints. Therefore, there is a strong need to embed dental sleep medicine, particularly in managing OSAS and related disorders, more deeply into dental curricula and postgraduate programs. Ongoing interdisciplinary collaboration and rigorous research are essential to critically evaluate and optimize emerging technologies, ensuring the advancement of evidence-based practices and improving patient outcomes in dental sleep medicine [66].

### 5.6. Clinical Implications

Despite these technological breakthroughs, significant barriers hinder effective clinical integration, including gaps in training for dental practitioners, inconsistent regulatory frameworks, and limited standardization of novel technologies. Current practices vary widely in the implementation and clinical validation of emerging devices, indicating the need for comprehensive clinical guidelines and consensus-driven protocols. Future research must focus on large-scale validation studies, rigorous evaluation of long-term outcomes, and developing standardized educational curricula to prepare dental professionals adequately [67,68]. Addressing these challenges will ensure the effective translation of technological advancements into meaningful improvements in patient care and interdisciplinary collaboration in dental sleep medicine.

## 6. Impact of OSAS Treatment on Oral Health

Treatment for OSAS, whether through CPAP or OAT, can have notable effects on oral health; however, serious or permanent complications are rare with appropriate monitoring. The most frequently reported impact is xerostomia (dry mouth), which is associated with both CPAP and oral appliances and can elevate the risk of dental caries and general oral discomfort; fortunately, this side effect is often mild and may improve with ongoing therapy or adherence to recommended practices [6]. Hypersalivation and drooling are also observed, particularly with MADs; however, these symptoms tend to be temporary and most pronounced at the initiation of therapy [5,69]. Some patients may experience tooth pain, muscle soreness, or mild jaw discomfort during the early stages of OAT.

Long-term OAT, commonly used to manage OSAS, can result in gradual occlusal changes, including decreased overbite and overjet, retroclination of upper incisors, and proclination of lower incisors [70]. However, regular dental monitoring generally ensures these alterations remain mild. Studies consistently indicate minimal impact on jaw mobility or function within the first year of appliance use, with a low risk of significant temporomandibular dysfunction. Regarding periodontal health, current evidence suggests that oral appliances do not adversely affect gums in patients without pre-existing periodontitis; nonetheless, close monitoring remains advisable for individuals with existing periodontal conditions [7,71,72].

To proactively mitigate these oral side effects, dental professionals should implement standardized clinical protocols. These should include regular follow-up visits every six months during the initial year of treatment and annually thereafter, involving comprehensive oral health evaluations, occlusal assessments, and periodic imaging. Patient education on optimal oral hygiene practices, including regular fluoride treatments and saliva substitutes, is critical to managing xerostomia-related risks [73]. Additionally, gradual titration of appliance advancement, timely appliance adjustments, and adjunctive therapies like physiotherapy or myofunctional therapy can effectively address persistent jaw discomfort, muscle pain, or occlusal disturbances [74,75].

### Clinical Implications

While OAT and CPAP remain cornerstone treatments for obstructive sleep apnea syndrome, their impact on oral health requires vigilant attention. Even though the dental and occlusal changes associated with prolonged OAT use are generally mild to moderate, these alterations reinforce the necessity for systematic dental follow-up and the establishment of clear clinical management pathways. At present, the available literature on the prevention and management of these oral side effects is limited in both scope and methodological rigor [76]. This paucity of high-quality evidence highlights an urgent need for the development of standardized monitoring protocols, as well as for educational initiatives that empower dentists to deliver targeted patient counseling and early interventions. Longitudinal research is particularly warranted to clarify the natural history of these complications and to identify the most effective strategies for safeguarding oral health in patients undergoing long-term OSAS therapy.

## 7. Interdisciplinary Collaboration

Interdisciplinary collaboration is increasingly recognized as a cornerstone of effective diagnosis and management of sleep apnea, as it enables the integration of expertise from a diverse array of healthcare disciplines, ultimately resulting in more comprehensive, individualized, and successful patient care, particularly for complex or persistent cases. Core team members within these collaborative models commonly comprise sleep physicians, dentists, and otolaryngologists (ENT), pulmonologists, pediatricians, and orofacial myologists, each of whom contributes unique skills to the evaluation and management process [77,78].

In more complex or pediatric cases, the interdisciplinary team may expand to involve nutritionists, myofunctional therapists, and other specialists, further enriching the treatment landscape. Importantly, while multidisciplinary care involves parallel contributions from various experts, true interdisciplinary care emphasizes integrated, collaborative decision-making and shared treatment planning that centers on the patient’s needs. These integrated models have been shown to enhance communication, streamline diagnostic processes, and improve treatment outcomes, particularly for pediatric OSAS or patients with comorbidities and persistent symptoms following standard therapies [79,80]. The use of standardized diagnostic tools, including the Pediatric Obstructive Sleep Apnea Diagnostic Examination Form (POSASDEF), facilitates effective communication and shared assessment between dental and medical professionals, while collaborative interventions, including the combined implementation of weight loss strategies, lifestyle modifications, and medical therapies, have been demonstrated to improve clinical outcomes and even reduce the necessity for CPAP in select patients [80]. Despite the clear benefits, challenges persist, including differences in training, communication barriers, and the lack of standardized protocols, which can impede optimal collaboration. Nonetheless, ongoing initiatives, including professional summits and consensus statements, are actively working to address these challenges and further promote interdisciplinary cooperation [81,82].

In summary, interdisciplinary collaboration leverages the collective expertise of diverse healthcare providers to yield more accurate diagnoses, tailored treatment plans, and superior outcomes for patients with sleep apnea, with integrated care models proving particularly valuable in managing complex or pediatric cases.

## 8. Dentists and Sleep Apnea in Low- and Middle-Income Countries

In low- and middle-income countries (LMICs), where underdiagnosis of sleep apnea syndrome remains a significant challenge, dentists are strategically positioned to serve as frontline sentinels for early detection and referral. During routine dental examinations, dentists are often the first healthcare professionals to observe signs indicative of OSAS, including craniofacial abnormalities, enlarged tongue, or evidence of airway obstruction [41,82]. Telehealth applications, including smartphone-based screening apps utilizing validated questionnaires (e.g., STOP-BANG), can facilitate rapid risk assessments without the need for extensive resources. Open-source educational platforms, virtual training modules, and webinars can enhance knowledge dissemination, allowing dentists to receive up-to-date training in sleep medicine at minimal cost [10,83]. This early identification is particularly critical, as untreated OSAS is associated with serious health risks, including hypertension, cardiovascular disease, and increased risk of accidents.

Despite their pivotal role, the effectiveness of dentists in LMICs is often limited by insufficient knowledge and negative attitudes toward sleep apnea, as documented in several studies, which underscores the urgent need for targeted educational and training initiatives [84,85]. Furthermore, there is considerable variability in dentists’ familiarity with diagnostic standards like polysomnography and available treatment modalities, reinforcing the necessity for enhanced professional development in this area. Optimal management of sleep apnea in resource-limited settings requires a multidisciplinary approach, with dentists actively collaborating with sleep specialists and other healthcare providers to ensure comprehensive care [80,86]. Additionally, low-cost diagnostic aids, including portable pulse oximeters and smartphone-compatible devices, could further empower dentists in preliminary screenings and patient monitoring, significantly improving early detection and interdisciplinary collaboration even in resource-constrained settings [87,88,89]. By integrating into multidisciplinary teams, dentists can contribute not only to the screening and referral process but also to the provision of treatment, including oral appliance therapy, thereby helping to bridge the gap in sleep apnea diagnosis and management in LMICs. Strengthening education, training, and collaborative practices among dental professionals is therefore essential to improve early detection and outcomes for patients in these regions.

### Clinical Implications

Despite the strategic role dentists can play in addressing OSAS in LMICs, substantial barriers remain, including limited awareness, inadequate training, and scarce resources. Current educational initiatives often lack sustainability, failing to create lasting improvements in clinical practice. Furthermore, the inconsistent availability and affordability of diagnostic tools and oral appliances hinder effective implementation of dental sleep medicine [53]. Addressing these challenges necessitates targeted investments in accessible training programs, affordable diagnostic technologies, and structured multidisciplinary collaborations. Future research should focus on evaluating cost-effective interventions and developing standardized protocols tailored to resource-constrained settings, thereby ensuring that dental professionals can meaningfully contribute to improving OSAS management and patient outcomes in LMICs.

## 9. Limitations

The evidence presented in this review primarily originates from observational and cross-sectional studies, inherently limiting causal conclusions about the intricate relationship between sleep apnea and oral health. Current literature demonstrates significant heterogeneity in diagnostic methodologies, screening practices, and referral protocols across clinical settings, thereby affecting generalizability. Additionally, most studies have relied on limited sample sizes or single-center designs rather than large-scale, multicenter randomized controlled trials, resulting in data fragmentation and variability in clinical outcomes reporting. Furthermore, the oral side effects associated with OSAS treatments remain inadequately characterized, inconsistently monitored, and often underreported in clinical practice, highlighting the critical need for standardized reporting guidelines.

In addition to the limitations already stated, much of the available evidence is of low to moderate methodological quality, often stemming from observational or cross-sectional studies with small sample sizes and high heterogeneity in methodologies. This restricts the strength of our conclusions and generalizability. While we have declared no conflicts of interest, we acknowledge the importance of ongoing transparency and encourage further independent research in this field.

## 10. Future Directions

To overcome existing methodological limitations and advance the field beyond current knowledge, future research should prioritize the design and execution of large-scale, multicenter randomized controlled trials (RCTs) focusing on interdisciplinary approaches involving dentistry and sleep medicine. The validation and clinical implementation of innovative screening tools tailored specifically for dental practice, especially practical solutions for resource-limited settings, should be emphasized. Emerging digital technologies, including AI-driven screening algorithms, three-dimensional craniofacial imaging, and wearable devices for continuous sleep monitoring, represent novel methodological frontiers warranting rigorous evaluation for clinical effectiveness and accuracy. Additionally, telehealth and cloud-based patient management platforms should be systematically assessed for their potential to enhance accessibility and interdisciplinary collaboration. Future studies must also incorporate longitudinal follow-up to comprehensively evaluate long-term dental, occlusal, and skeletal impacts of oral appliance therapy, alongside patient adherence patterns and quality-of-life metrics.

Finally, addressing current knowledge gaps necessitates targeted development of innovative interprofessional educational programs. Digital education tools, including virtual-reality and simulation-based platforms, offer promising novel pathways for enhancing dentist training, interdisciplinary teamwork, and evidence-driven patient care, especially in underserved and resource-constrained regions. These integrated, technology-enhanced approaches have substantial potential to establish more consistent clinical standards, significantly advancing both the quality of research and patient outcomes in dental sleep medicine.

We propose the establishment of multicenter and international collaborations to standardize protocols, share data, and strengthen evidence regarding dental approaches to sleep apnea. These initiatives could enable large-scale, comparative studies and foster interdisciplinary research networks.

## 11. Conclusions

Dentists play a pivotal role in the early detection, referral, and management of obstructive sleep apnea syndrome, particularly given their unique ability to identify oral and craniofacial risk factors. Strengthening their role through targeted education, standardized protocols, and enhanced collaboration with sleep specialists is essential to improve patient outcomes.

As dental sleep medicine evolves, integrating dentists more fully into multidisciplinary care teams can address persistent gaps in diagnosis and management, especially in resource-limited settings, ultimately improving the quality of life and health of patients with sleep apnea syndrome.

## Data Availability

The data presented in this study are available upon request from the corresponding author.
